# Management of triple negative breast cancer in a centenarian

**DOI:** 10.1002/cnr2.1642

**Published:** 2022-06-02

**Authors:** Andrew Donaldson, Lilia Lunt, Andrea Madrigrano

**Affiliations:** ^1^ Department of Surgery Rush University Medical Center Chicago Illinois USA; ^2^ Division of Surgical Oncology, Department of Surgery Rush University Medical Center Chicago Illinois USA

**Keywords:** advanced age, centenarian, elderly, triple‐negative breast cancer

## Abstract

**Background:**

There is limited clinical data to guide treatment for elderly patients with triple‐negative breast cancer (TNBC). In the case of centenarians, there is almost no data for this age group. The diagnosis of TNBC portends a more challenging clinical course compared to hormone receptor positive breast cancers, especially in elderly patients.

**Case:**

We present the case of a 102‐year‐old patient who was diagnosed with TNBC. Although our initial plan was observation, the tumor growth rate and the pain it caused resulted in us offering a right total mastectomy and a left partial mastectomy.

**Conclusion:**

Morbidity and mortality are higher in TNBC patients, and treatments are more limited, especially in elderly patients who may not be able to tolerate chemotherapy or surgery. As a result, management of breast cancer in elderly patients is largely individualized and treatment is generally more conservative. Focusing on quality of life is a key consideration when treating this patient population.

## 1 INTRODUCTION

Triple‐negative breast cancer (TNBC) represents approximately 11%–17% of female breast cancers and is primarily a cancer of younger women, with some studies showing highest incidence in women <40 years old.^1^, ^2^, ^3^, ^4^, ^5^ TNBC is a subset of breast cancer that lacks expression of the estrogen receptor (ER), progesterone receptor (PR), and the human epidermal growth factor receptor 2 (HER2). Compared to hormone receptor positive breast cancer, TNBCs are generally categorized as higher‐grade, can exhibit rapid growth, and have a higher risk of local and distant recurrence.^2^, ^5^ As a result, TNBCs are often associated with a worse prognosis.^3^, ^5^ As opposed to hormone receptor positive breast cancers, patients with TNBC are not candidates for targeted endocrine therapies such as tamoxifen or trastuzumab.^1^, ^4^ The treatment paradigm for TNBC depends on a multitude of factors, but generally involves a combination of surgery and chemotherapy.^2^, ^3^, ^5^, ^6^ In elderly patients, treatment of TNBC is often more conservative, owing to concerns of tolerability of systemic therapies. Studies have demonstrated that, compared to younger patients, elderly patients are less likely to undergo or complete chemotherapy.^4^, ^7^, ^8^, ^9^

We present the case of a rapidly growing triple negative invasive ductal carcinoma (IDC) that was diagnosed and treated in a 102‐year‐old patient. The patient's advanced age prompted numerous considerations that had a significant impact on her care. Our approach was focused on improving quality rather than quantity of life. Ultimately, the aggressiveness of the tumor and the patient's resulting pain informed our decision to offer surgery.

### 1.1 Case description

The patient is a 102‐year‐old female who was referred to the Rush University Cancer Center for evaluation of a palpated right breast mass. Her medical history was significant for hypertension, hypercholesterolemia, chronic kidney disease, osteoporosis, Vitamin D deficiency, and frequent mechanical falls. Despite her comorbidities and age, the patient still led a very independent life and was performing her activities of daily living without issue. We graded her Eastern Cooperative Oncology Group (ECOG) status as one. There was no personal or family history of breast or ovarian cancer and the patient had no prior breast surgeries or biopsies.

**FIGURE 1:**
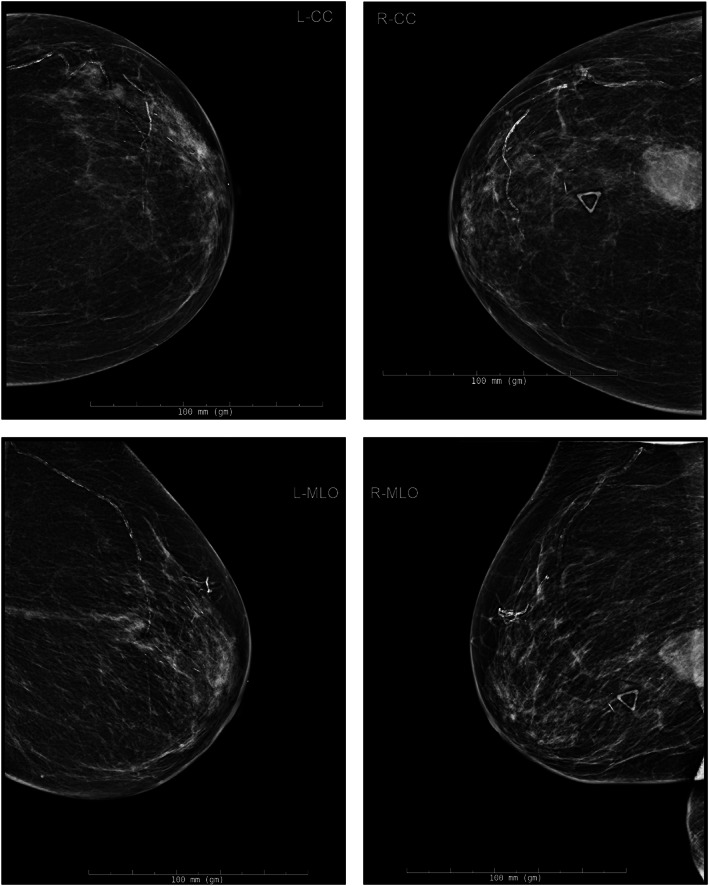
Bilateral diagnostic mammogram at the time of initial evaluation. Top left: left breast, cranio‐caudal view. Top right: right breast, cranio‐caudal view. Bottom left: left breast, mediolateral oblique view. Bottom right: right breast, mediolateral oblique view

On initial physical examination, we palpated bilateral breast masses without skin changes. A bilateral diagnostic mammogram demonstrated a right breast mass measuring 2.5 × 2.4 × 2.6 cm and two adjacent left breast masses measuring 2.4 × 1.0 × 0.9 cm in total, BIRAD 4C (Figure 1). Ultrasound of the bilateral axillae demonstrated no pathologically appearing lymph nodes. Based on the clinical and radiographic findings, we shared with the patient and her daughter our concern that these masses represented malignancies. We offered three options–observation, core biopsy, or upfront excision–although the patient expressed a desire to avoid any sort of intervention if possible.

We subsequently discussed the case at our multidisciplinary tumor board. Given the patient's age, the primary focus of treatment would be symptom control. The tumor board ultimately did not recommend systemic staging, as any positive findings would not have changed our treatment recommendation. Furthermore, if a biopsy demonstrated ER‐positive cancer, the patient would not be a candidate for endocrine therapy given her profound osteoporosis and frequent falls. We shared this consensus with the patient and her daughter, who agreed with this approach and opted for short‐term follow up to assess stability. They understood that tumor growth may necessitate intervention.

**FIGURE 2:**
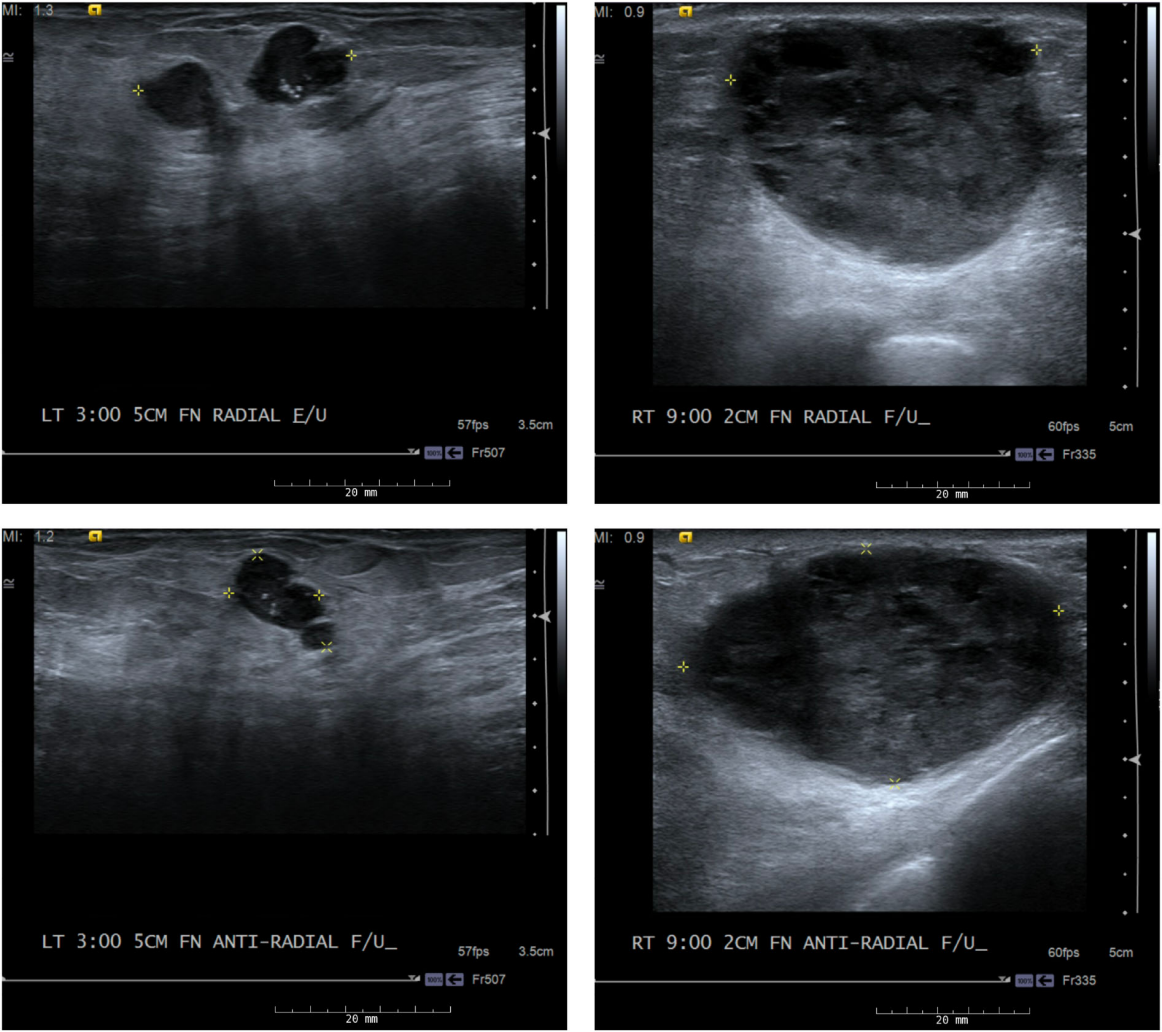
Follow‐up bilateral breast ultrasound images, 11 weeks from initial diagnostic mammogram. Top left: left breast, radial. Top right: right breast, radial. Bottom left: left breast, antiradial. Bottom right: right breast, antiradial

Over the course of 11 weeks, the right breast mass rapidly increased in size. Follow‐up ultrasound indicated the mass had grown to 5.0 × 3.1 × 4.0 cm, BIRAD 5 (Figure 2). The left breast mass demonstrated no significant change in size. Based on these findings, we again determined that a preoperative biopsy was very unlikely to change our course of action. We recommended resection with the primary goal to improve quality of life as these masses were now causing the patient significant pain.

Surgery was performed 3 weeks following her second ultrasound; including the 11‐week observation period, this was 3 months after the initial consultation. At this time, we noted that the right breast mass had markedly increased in size. The mass occupied the majority of the breast and caused significant skin dimpling. The patient was taken to the operating room and induced under general anesthesia without complication. We performed a right total mastectomy and a left partial mastectomy, which were well‐tolerated. Given the patient's age, she was observed in the hospital for 23 h where she made an uneventful recovery. Her pain was well‐controlled without the use of opiates and she was discharged home to the care of her daughter.

Pathology of all specimens was IDC, grade 3, pT3Nx for the right mass and pT2Nx for the left mass, without skin involvement. The right breast mass measured 5.1 cm in greatest dimension and the left breast mass measured 2.2 cm in greatest dimension. Immunohistochemical staining of both masses was performed. The right breast mass was ER/PR negative (<1%), HER2 2+, and Ki‐67 > 20%. Fluorescence in situ hybridization demonstrated the right breast mass to be HER2 negative. Similarly, the left breast mass was ER/PR negative, HER2 0+, and Ki‐67 > 20%.

At the first postoperative clinic visit, the patient was making a normal recovery and had no complaints. Pain was well‐controlled with acetaminophen alone. The pathology was discussed with the patient and her daughter. The patient was offered referrals to medical and radiation oncology for consultation regarding the possibility of adjuvant treatment, which she declined. She was to return to our clinic for ongoing postoperative surveillance. At her last clinic visit–ten months postoperative–the patient was in good health and did not have any complaints. She had returned to her baseline daily activities, still lived independently, and had no evidence of recurrent disease. She had not received any adjuvant therapy.

## 2 DISCUSSION

We presented the case of triple‐negative IDC in a centenarian with a particularly aggressive primary tumor. Many aspects of this patient's case are unique, and they shed light on important topics related to cancer care for older patients. Most notable is the patient's advanced age. Initial presentation of TNBC in this age range is abnormal, as it most commonly presents in younger women.^1^, ^2^, ^3^ To our knowledge, management details of TNBC for a patient of this age have not been reported in the literature, yet it was her age that most profoundly influenced our goals of treatment. The impressive growth rate of the tumor–having increased in size approximately four‐fold in 14 weeks–also played a key role in our decision making, as it had begun causing constant pain. Our dilemma then became balancing the patient's quality of life with the increased risk of morbidity and mortality of operating on someone in her age group.^10^

We ultimately offered treatment to alleviate symptoms and maintain quality of life rather than to extend it. This entailed performing a mastectomy but no systemic treatment. We acknowledge that this approach **is** unconventional, especially considering that our consensus was to exclude a preoperative staging workup. When discussing the standard preoperative staging process, it became apparent that the patient desired the minimum amount of intervention (surgery or chemotherapy), regardless of any additional findings outside the breast. Given her lack of symptoms at that time, close observation seemed appropriate. However, when the patient's breast masses grew and began causing constant pain during the observation period, we determined that a preoperative biopsy would not change the fact that resection was the best and least invasive approach to alleviating her symptoms.

Caring for a 102‐year‐old patient highlighted some salient points. First of all, we found that there is very little data in the literature to guide breast cancer treatment for this age group, despite the expanding population of patients ≥75 years old.^9^, ^11^, ^12^, ^13^ In fact, there is insufficient evidence for the NCCN to recommend chemotherapy regimens in breast cancer patients older than 70 years.^6^ As a result, elderly breast cancer patients generally seem to be offered more conservative treatment regimens, which in some circumstances could be considered undertreatment.^13^ Some retrospective studies have demonstrated that elderly patients with TNBC are less likely to have an oncology consultation, less likely to be offered/complete chemotherapy, and more likely to be treated with surgery and radiation alone.^4^, ^7^, ^8^, ^9^ Other studies highlight the unique decisions providers must address for this population when approaching treatment, similar to those that we considered in this case. Specifically, factors such as overall treatment goal (survival versus palliation), life expectancy, treatment toxicity and tolerance, and the patient's personal goals should all receive attention.^8^, ^9^ Such was the case in two similar reports of elderly patients with breast cancer, which focused on the balance of treatment risk/tolerance and quality of life.^14^, ^15^

It should be noted that there is an increasing body of knowledge that contradicts the belief that advanced age independently incurs an increased risk of perioperative morbidity and mortality. Other metrics, such as frailty assessments, may be more accurate than age alone in determining operative risk in the elderly. This is a topic of ongoing investigation.

## 3 CONCLUSION

The management of breast cancer in elderly patients demands consideration of the overall goal of treatment and what treatments the patient would be able to tolerate. This point is even more pertinent to centenarians, for whom there is very limited data regarding operative risk and administration of systemic therapy to guide clinical decision making. Our focus was the patient's quality of life, and she ultimately had a positive outcome. It has now been over 15 months since our patient's surgery; she is currently without evidence of disease and continues to live independently without impairment of her activities of daily living.

## AUTHOR CONTRIBUTIONS

**Andrew Donaldson:** Conceptualization (equal); writing – original draft (lead); writing – review and editing (equal). **Lilia Lunt:** Conceptualization (equal); writing – original draft (equal); writing – review and editing (equal). **Andrea Madrigrano:** Conceptualization (equal); supervision (lead); writing – review and editing (equal).

## CONFLICT OF INTEREST

All listed authors have declared that they have no conflicts of interest related to the content of this case report.

### ETHICS STATEMENT

Rush University Medical Center Institutional Review Board has determined this project to be exempt from IRB oversight, as it does not constitute human subject research. Written informed consent was obtained from the patient for the publication of case details and the use of images.

## Data Availability

Data sharing is not applicable to this article as no new data were created or analyzed in this case report.
